# Supplementary material for: Multidimensional scaling methods can reconstruct genomic DNA loops using Hi-C data properties
Source: PLoS One. 2023 Aug 17;18(8):e0289651. doi: 10.1371/journal.pone.0289651 (PMC10434948; doi:10.1371/journal.pone.0289651)
Supplement: Attachment — Submitted filename: Response to Reviewers.pdf [file pone.0289651.s002.pdf]

1. In the main text, the author uses "ChIP-seq" and shows a table of the analysis of H3K27ac, which is related to the transcriptionally active state. However, the author did not mention the chromatin state or motivation to analyze ChIP-seq data. The author should mention such information.

A.)

The following additions were made as to why the ChiP-seq and ATAC-seq data were analysed.

L205:'Chromatin immunoprecipitation followed by sequencing (ChIP-seq) is an experimental technique for mapping DNA-binding proteins and histone modifications [26]. Assay for Transposase-Accessible Chromatin with high-throughput sequencing (ATAC-seq) investigates DNA accessibility using hyperactive Tn5 transposase [27]. ATAC-seq helps identify open chromatin regions and assess transcription factor occupancy. DNA loops have many arbitrary protein interventions to promote or regulate transcription, and three spatial spaciousness, so selected DNA loops and ChIP-seq and ATAC-seq should match the peaks in the data. Therefore, the DNA loop regions selected by the MDS-based method were rechecked to determine whether they matched the locations of ChIP-seq or ATAC-seq peaks. When a partial match was found between the position of the top 300 peaks and the DNA loop regions selected by the MDS-based method, the loop was counted. Fisher's exact probability test was then performed to determine whether the selected DNA loops significantly matched the peaks compared to randomly selected loops.'

2. How did the author find the logarithmic weight in Eq. (1)? What is the base of the logarithm? If the base is 5, the equations should make sense.

A.)

It is the base e of the natural logarithm. Indeed, 50,000 bp was used as a cut-off value, but it is unnecessary to set the base of the logarithm to 5. Hi-C data with distances between genomic coordinates of less than 50,000 bp were not changed because the values are large by default. The only important pre-processing was to reduce the gap by multiplying equation (A) by the Hi-C contact frequency, although the distances are far apart. This part needed to be explained better, so the following additions were made. Also, the word "log" has been corrected to "$\log_e$" to avoid misunderstandings.

L110: 'Hi-C contact frequency between distant coordinates that form DNA loops are underestimated compared to Hi-C contact frequency between initially close coordinates [7]. The only important pre-processing step was to reduce the gap between Hi-C contact frequencies using the multiplying equation (1).'

3. The base of the logarithm of the Y-axis in Figs. 1 and 2 should be described.

A.)

In the title of Figs 2 and 3, it was corrected to 'natural logarithm' instead of 'logarithm'.

Fig2: Plot of the distance between coordinates versus the natural logarithm of Hi-C contact frequency before multiplying by weight

Fig3: Plot of the distance between coordinates versus the natural logarithm of Hi-C contact frequency after multiplying by weight

4. The reviewer could not understand the data format in Supplementary Files. For example, the file "GSE141067(osteosarcoma)/0min/SVD_234/plot.chr1.0-4980.txt" has 5 columns and 2989 rows.

A.)

The data format has been improved. The data is a three-column data frame with SVD eigenvectors $\mathbf{v}_2$, $\mathbf{v}_3$, and $\mathbf{v}_4$.

5. l. 130 and Eq. (3): The author converted the contact frequency into the distance without any references. Although the assumption is not theoretically supported in terms of polymer physics [Shinkai et al. NARGAB 2020], some computational methods have conventionally assumed [Serra et al. FEBS Lett 2015].

A.)

A citation is added for the adoption of the inverse of the interaction frequency. A polymer model such as PHi-C was not used because it is highly dependent on the performance of the Hi-C data.

L139: 'PHi-C' is a method to reproduce genome structure by considering a simple macromolecular model of the genome (a single chromosome) as a 'linked bead' [21]. PHi-C accurately estimates genome structure in four dimensions but requires dense Hi-C data. In this case, a versatile model was desired, so the reciprocal frequency of interactions between two particles in the model (i.e., between two bins or restriction fragments in the genome) was adopted as the distance. The inverse of the power of the frequency is debatable, but it was set to 1 as used in fractal globule models [22].

6. Fig. 3 legend must be incomplete. The author only plotted the eigenvectors. Moreover, the figure includes blue lines without explanation.

A.)

The title of Figure 4 was changed to "Plot of the second and third eigenvectors". The blue line represents the root of the DNA loop. The definition of a DNA loop is described in Section 2.2.4. The following text was added to the legend in Figure 4.

Fig4: 'The blue lines represent the root of the DNA loop, which is defined in Section 2.2.4.'

7. What does the author mean by "roots" in Fig. 4 legend?

A.)

This implies a root in the DNA loop. In other words, it is an enhancer or promoter.

The description of the selection criteria for DNA loops was unclear and has been corrected as follows:

L191: Formulaically, let T be the threshold, and $\mathbf{E}_i'$ be the distance between genomic coordinates, where i is the coordinates divided by the Hi-C resolution. Moreover, n_k is the coordinate such that $\mathbf{E}'_{nk} - \mathbf{E}'_{nk-1} < 0$ and $0 < \mathbf{E}'_{nk+1} - \mathbf{E}'_{nk}$. Then, for any i, n'_k is defined as the n_k with $n_k < i < n_{k+1}$ that satisfies $\mathbf{E}_i' < T$. Finally, the DNA loop was obtained from n'_{k-1} to n'_{k+1}.

→

Formulaically, let T be the threshold, $\mathbf{E}_i'$ greater than the threshold T is denoted as $\mathbf{E}^+_i$ and is a DNA loop, where i is the coordinates divided by the Hi-C resolution. On the other hand, $\mathbf{E}_i'$ smaller than the threshold value is denoted as $\mathbf{E}^-_i$. When proceeding from the both sides of DNA loop $\mathbf{E}^+_i$ to directions i+j and i-j (j is any natural number) that are smaller than the threshold value T, the root of the DNA loop is defined as $\mathbf{E}^-_i - \mathbf{E}^-_{i-j} < 0$ and $0 < \mathbf{E}^-_{i+j} - \mathbf{E}^-_I$.

8. Eq. (7): The variable "V" seems to be a new one. What is the difference between "v" and "V"?

A.)

'V' is the unitary matrix of the singular value decomposition and '**v**' is its column vector. Equation 7 has been modified because 'V' and '**v**' have been mixed.

Equation 7 : $E_i = \sqrt{(\mathbf{V}_{i+1,2} - \mathbf{V}_{i,2})^2 + (\mathbf{V}_{i+1,3} - \mathbf{V}_{i,3})^2}$

$\rightarrow E_i = \sqrt{(\mathbf{v}_{i+1,2} - \mathbf{v}_{i,2})^2 + (\mathbf{v}_{i+1,3} - \mathbf{v}_{i,3})^2}$

L164: 'V is an orthogonal matrix'→'V is an unitary matrix'

L166:'Empirically, the second and third eigenvectors **V** after eigenvalue decomposition frequently retain their original structure.'

→'Empirically, the second and third eigenvectors $\mathbf{v}_2$, $\mathbf{v}_3$ after eigenvalue decomposition frequently retain their original structure.'

9. l. 174-183: Can this algorithm uniquely determine loop regions? This definition must be the most critical part of this work. However, the reviewer could not understand it and Fig. 4 correctly. A schematic of the algorithm would help broad readers.

A.)

As MDS is reproducible, DNA loops can be uniquely determined, like singular value decomposition. However, it depends on how many parts of the Hi-C data are split. In this study, from the point of view of the computational complexity of the multidimensional scaling construction method, the Hi-C data were split into 1 to 5 pieces for analysis.

The description of the selection criteria for DNA loops was unclear and has been corrected as follows:

L191: Formulaically, let T be the threshold, and $\mathbf{E_i'}$ be the distance between genomic coordinates, where i is the coordinates divided by the Hi-C resolution. Moreover, n_k is the coordinate such that $\mathbf{E'_{nk}} - \mathbf{E'_{nk-1}} < 0$ and $0 < \mathbf{E'_{nk+1}} - \mathbf{E'_{nk}}$. Then, for any i, n'_k is defined as the n_k with $n_k < i < n_{k+1}$ that satisfies $\mathbf{E_i'} < T$. Finally, the DNA loop was obtained from n'_{k-1} to n'_{k+1}.

→

Formulaically, let T be the threshold, $\mathbf{E_i'}$ greater than the threshold T is denoted as $\mathbf{E_i}^+$ and is a DNA loop, where i is the coordinates divided by the Hi-C resolution. On the other hand, $\mathbf{E_i'}$ smaller than the threshold value is denoted as $\mathbf{E_i}^-$. When proceeding from the both sides of DNA loop $\mathbf{E_i}^+$ to directions i+j and i-j (j is any natural number) that are smaller than the threshold value T, the root of the DNA loop is defined as $\mathbf{E_i}^- - \mathbf{E_{i-1}}^- < 0$ and $0 < \mathbf{E_{i+1}}^- - \mathbf{E_i}^-$.

The following flowchart of the algorithm has been added.

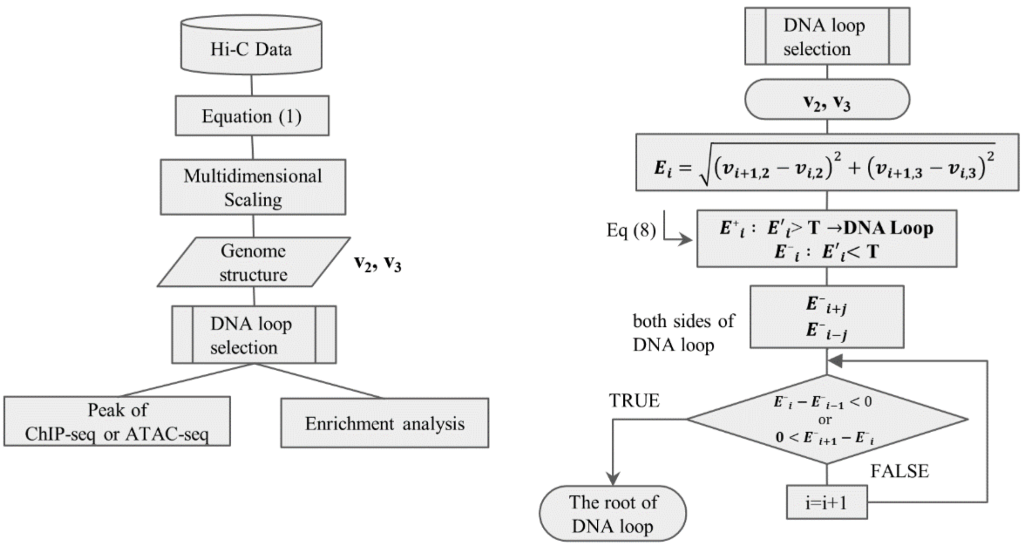

Fig 1. The flowchart of analyses performed in this study

10. p. 6: Please remove the Fig.5 legend.

A.)

Legend in Fig 6 was eliminated as follows.

Fig.6 **Distance plot between coordinates of chromosome 5 50,000-100,000 kbp of series GSM6061774 by the MDS-based method.** (The red line is the threshold).

→Fig.6 **Distance plot between coordinates of chromosome 5 50,000-100,000 kbp of series GSM6061774 by the MDS-based method.**

11. l. 207: The author describes, "The number of DNA loops and number of genes are close to each other," referring to Table 1. However, the number of DNA loops is almost 30000, and the number of genes is almost 5000 in Table 1. These are NOT close to each other.

A.)

I wrote that 'The number of DNA loops and number of genes are close to each other', but this was a mistake. The number of DNA loops is almost the same for replicates, and the number of genes is almost the same for replicates. The following has been corrected.

L233: 'The number of DNA loops and number of genes are close to each other',

→'the number of DNA loops is almost 10%, and the number of genes is almost 22%.'

12. § 3.2: Has the author analyzed H3K27me3 ChIP-seq data? The histone marker is related to heterochromatin.

A.)

H3K27me3 is involved in heterochromatin but is also found to be abundant in DNA loops and represses gene expression. Therefore, I analyzed the H3K27me3 ChIP-seq data and found that the positions of the peaks in the H3K27me3ChIP-seq data and CTCF and H3K27ac significantly coincided with the positions of the selected DNA loops.

L219: 'ChIP-seq data for H3K27ac, H3K27me3, and CTCF were analyzed; H3K27ac is an active enhancer

marker, CTCF is involved in DNA loops, H3K27me3 is involved in heterochromatin but is also found to be abundant in DNA loops and represses gene expression [28].'

13. Figure 7 is not attached to the manuscript.
A.)
Unnecessary latex codes were listed and misleading (Fig5). It has been deleted.

Reviewer #2: In this manuscript, Ryo utilizes multidimensional scaling (MDS) to capture DNA loops for high-throughput chromosome conformation capture (HiC) data. By transforming HiC data to a distance matrix, MDS identified DNA loops contained more DNA bound by transcription factors than existing methods. The manuscript is well organized.

I have 4 major concerns.

1. In section 2.2.2, the term "exaggerated representations of DNA loops" is confusing. Could you clarify the definition and rationale behind it? I am also concerned about the threshold of 50kbp for formula 1. Would the significant loops of length 50kbp be affected by this transformation?

A.)
"Exaggerated representations of DNA loops" was confusing. Therefore, instead of "exaggerated representations of DNA loops", the term "preprocessing to clearly represent DNA loops" was used.
'Clear representations of DNA loops' means that the Hi-C data is transformed using equation (1), and MDS is applied to reproduce a genome structure that shows DNA loops more clearly than if no pre-processing is applied to the Hi-C data.

In equation (1), 50 kb was set as the cut-off value. No extra processing was added to the Hi-C contact frequencies within 50 kb of the genomic co-ordinates, as these are large by default; for Hi-C contact frequencies above 50 kb, the gap in the Hi-C data was filled by multiplying by the natural logarithm of the distance. For example, changing the cut-off value of 50 kb to 70 kb does not significantly change the structure of the genome. However, setting the cut-off to 20 kb or less will change the genome structure because the Hi-C contact frequency below 20 kb is smaller because of the natural logarithm. The present study found that setting the cut-off value to 50 kb reproduces the genome structure, which clearly represents the DNA loop, although there is no strict biological basis for this.

L121: For Hi-C contact frequencies above 50 kbp, the gap in the Hi-C data was filled by multiplying by the natural logarithm of the distance. For example, changing the cut-off value of 50 kbp to 70 kbp does not significantly change the structure of the genome. However, setting the cut-off to 20 kbp or less will alter the genome structure because a Hi-C contact frequency below 20 kbp will be low because of the natural logarithm. The present study found that setting the cut-off value of 50 kbp reproduces a genome structure that clearly represents the DNA loop, although there is no strict biological basis for this.

2. In section 2.2.3, the authors claimed that the second and the third eigenvectors often retain the original structure. Empirically, how would one decide for real HiC data which eigenvectors to use for identifying

loops? Why is the first eigenvector excluded?

A.)

Empirically, the second and third eigenvectors $\mathbf{v}_2$, $\mathbf{v}_3$ after eigenvalue decomposition frequently retain their original structure. This was verified in my previous paper with a simple simulation. Therefore, after eigenvalue decomposition, $\mathbf{v}_2$ and $\mathbf{v}_3$ of the eigenvector $\mathbf{V}$ were chosen as the genomic structure. In the following, two simple simulations were performed.

The column of the orthogonal matrix $\mathbf{U}$ presenting the desired structure is unclear. However, in many cases, the second and third columns of the orthogonal matrix $\mathbf{U}$ are thought to present the considering structure. the first one is 500 random numbers ranging from -100 to 100 and the second one is a circle composed of random numbers, such as $x_{i1} = \cos\left(2\pi\frac{i}{N}\right) + g(\lambda)$, $x_{i2} = \sin\left(2\pi\frac{i}{N}\right) + g(\lambda)$. Here, the function g is the error function and is defined as follows: $g(\lambda) = \lambda, 0 \leq \lambda \leq 0.5$

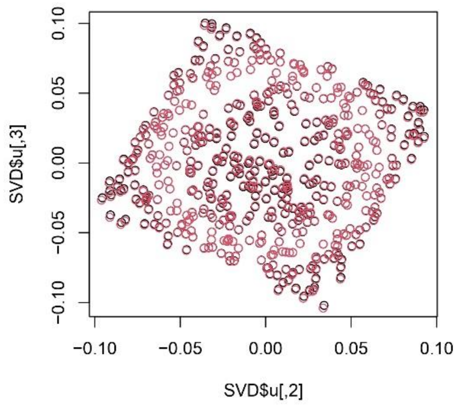

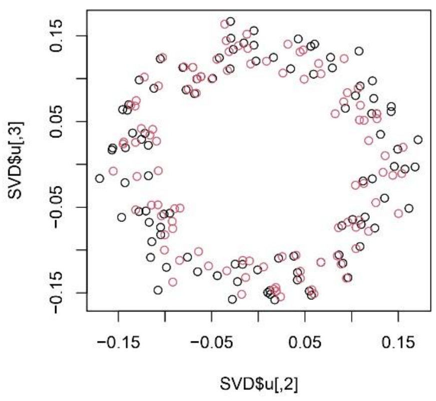

Fig. A Simulation 1 of the $\mathbf{v}_2$ and $\mathbf{v}_3$ eigenvector

Fig. B Simulation 2 of the $\mathbf{v}_2$ and $\mathbf{v}_3$ eigenvector

The black dots are the original coordinates, and the red are the v_2 and v_3 plots from MDS. As can be seen, by this simple simulation, it is empirically found that the second and third eigenvectors represent the original coordinates. Therefore, the second and third eigenvector after SVD was also adopted as the desired structure in this study.

3. In formula 8, the distance matrix is smoothed with a window size of 100kbp. Has the author evaluated the performance of MDS varying different window sizes? For other HiC data, how would the user recommend selecting this hyperparameter?

A.)

Smoothing is debatable. For example, 40kbp of smoothing worked well, and without smoothing, the loop could not be fully selected. Future research is needed to find a biologically valid smoothing. In this study, smoothing was performed at 10kbp, which is neither too long nor too short. Further investigation is needed when dealing with other resolutions.

L183: ‘This smoothing was performed at 100 kbp, which is neither too long nor too short. Further investigation is needed when dealing with other resolutions.’

4. In table 1, is 318380 the number of all potential DNA loops considered? What do the ratios in two columns

represent? # of loops seems much bigger than # of genes in table 1, which contradicts the statement in lines 207-210, “the number of loops and number of genes are close to each other?” Could you quantify this?
A.)
Fisher's exact probability test was performed to determine whether the enhancer-promoter regions in this study were significantly consistent with selected DNA loop regions compared to the randomly selected one. The following representation is misleading and has been revised.

L216: In the present study, Fisher's exact probability test was performed to determine whether the enhancer-promoter regions in this study were significantly consistent with random selections.
→Fisher's exact probability test was then performed to determine whether the selected DNA loops significantly matched the peaks compared to randomly selected loops.

In GSM6061774, 318,380,000 bp were selected as loop regions, of which 2,410,018 bp were partially matched to peaks of the ChIP-seq data. The expected value was determined by multiplying the proportion of the top 300 peak regions (peak regions divided by total genomic regions) by the length of the loop region. The p-value for Fisher's exact probability test was 2.2×10^{-16}, and the odds ratio was 39.33, indicating significant agreement.

L343: In GSM6061774, 318,380,000 bp were selected as loop regions, of which 2,410,018 bp were partially matched to peaks of the ChIP-seq data. The expected value was determined by multiplying the proportion of the top 300 peak regions (peak regions divided by total genomic regions) by the length of the loop region. The p-value for Fisher's exact probability test was 2.2×10^{-16}, and the odds ratio was 39.33, indicating significant agreement. Other data were also in significant agreement (Supplementary Files).

I wrote that ‘The number of DNA loops and number of genes are close to each other’, but this was a mistake. The number of DNA loops is almost the same for replicates, and the number of genes is almost the same for replicates. The following has been corrected.
L233: ‘The number of DNA loops and number of genes are close to each other’,
→’the number of DNA loops is almost 10%, and the number of genes is almost 22%.’

I have 3 minor comments.
1. It would be helpful to have a table of datasets to summarize the read depth, bin size, year, and HiC data type.
A.)
Information on the seven Hi-C datasets analyzed in this study is summarized in the Table1.
Table1. Information on the seven Hi-C datasets analyzed in this study

Series	Cell type	Read depth	Bin size [bp]	Year	Instrument
GSE201353	macrophages	-	10,000	2022	Illumina NovaSeq 6000
GSE141067	osteosarcoma	-	50,000	2020	Illumina NovaSeq 6000
GSE149103	pancreas	3 billion	10,000	2021	HiSeq X Ten
GSE160235	colorectal gland	450 million	10,000	2021	Illumina NovaSeq 6000
GSE167150	breast	600 million	10,000	2022	Illumina NovaSeq 6000
GSE168470	lymphoma	-	10,000	2021	Illumina HiSeq 2500 and Illumina NextSeq 500
GSE143465	kidney	3 billion	10,000	2021	Illumina NovaSeq 6000

2. Below line 195, the description of figure 5 should be revised.

A.)

Unnecessary latex codes were listed and misleading. It has been deleted.

3. The grammar and wording can be much improved for this manuscript.

A.)

This manuscript was proofread by editage.

Reviewer #3: Ishibashi suggested a method (MDS) to predict DNA loops based on Hi-C sequencing data. It tested on 7 publicly available Hi-C datasets from various cancers (e.g., breast cancer, lymphoma, and kidney cancer). However, there are four major problems in the manuscript:

1) In the Methods section, the description of MDS in predicting DNA loops is not so clear. The algorithm, code and demo data for this study shall be publicly available for others, in order to reproduce the results and understand the method. The prediction accuracy for DNA-loops has to be rigorously evaluated by proper statistical methods.

A.)

Algorithm flowchart added in Fig 1. Also, the code has been posted to GitHub. The results of this study were submitted to 'figshare' as a supplemental file, and the validation of the DNA loops was rigorously evaluated by Fisher's exact probability test.

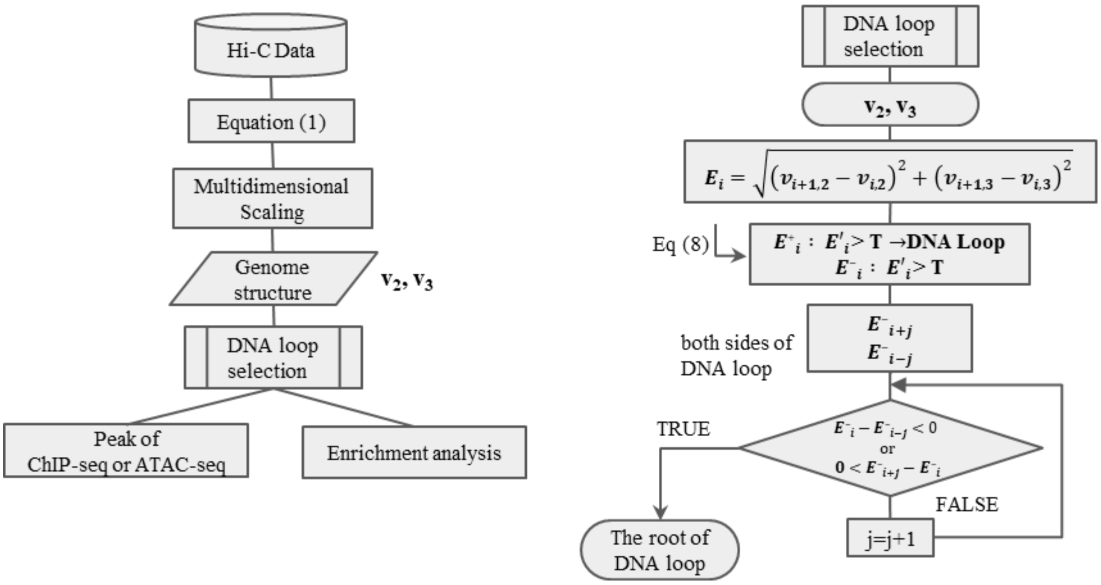

Fig 1. The flowchart of analyses performed in this study

L : R source code. A sample of the R source can be found at https://github.com/Ryo-RIshibashi/MDS_DNALoop.

The description of the selection criteria for DNA loops was unclear and has been corrected as follows:

L191: Formulaically, let T be the threshold, and **E_i’** be the distance between genomic coordinates, where i is the coordinates divided by the Hi-C resolution. Moreover, n_k is the coordinate such that **E'_{nk}- E'_{nk-1}** <0 and 0< **E'_{nk+1}- E'_{nk}**. Then, for any i, n'_k is defined as the n_k with n_k <i< n_{k+1} that satisfies **E_i’**<T. Finally, the DNA loop was obtained from n'_{k-1} to n'_{k+1}.

→

Formulaically, let T be the threshold, **E_i’** greater than the threshold T is denoted as **E_i^+** and is a DNA loop, where i is the coordinates divided by the Hi-C resolution. On the other hand, **E_i’** smaller than the threshold value is denoted as **E_i^-**. When proceeding from the both sides of DNA loop **E_i^+** to directions i+j and i-j (j is any natural number) that are smaller than the threshold value T, the root of the DNA loop is defined as **E_i^-** - **E_{i-1}^-** < 0 and 0 < **E_{i+1}^-** - **E_i^-**.

2) In the Results section, the presentation of predicted loops in each dataset is too short to evaluate its merit. In Particular, the results have to be compared with other similar tools for predicting DNA loops by using Hi-C sequencing datasets. And also, I am not clear about how other genomic information (e.g., enhancer or promoter histone markers and nucleosome density) at these predicted loops, which are important markers for validating the predicted DNA loops/interactions, are functional or random interactions.

A.)

This manuscript compares this method with my old method and miniMDS. In the results section, I mentioned that the present method gave better enrichment analysis results and more genes were included in the selected DNA loops. I tried another method, StoHi-C, which clearly outputs undesirable structures (supplemental files). All chromatin structures are shown in the supplemental file.

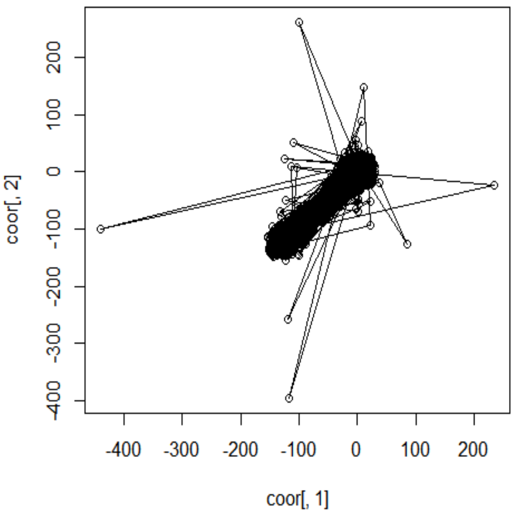

Fig S1. Genome Structure by stoHi-C (Chromosome5 0-50,000,000 bp)

3) In the Discussions section, the author needs to improve this part significantly to convince people that the proposed new method is useful for studying DNA loops based on Hi-C data.

A.)

The discussion section has been greatly improved to convince the reader.

L376: ‘One of the main goals of this study was to identify a method of reconstructing the genomic structure that differentiates DNA with and without loops. As indicated in the results, the validity of the proposed method is verified by the following three findings. (i) The number of genes was quite large for the region selected as the DNA loop. (ii) Transcription factors involved in DNA loop formation were enriched in the enrichment analysis by g: profiler. (iii) The positions of the selected DNA loop regions and the ChIP-seq and ATAC-seq peaks were significantly consistent. To my knowledge, no existing study has been able to confirm the consistency of the DNA loop to this extent. Therefore, since the consistency of (i)-(iii) was confirmed in a data-driven manner, this method is useful for reproducing DNA loops.’

4) Finally, the quality of all main figures is not good, which has to be improved significantly.

A.)

All figures have been improved.
