# Supplementary material for: Multidimensional scaling methods can reconstruct genomic DNA loops using Hi-C data properties
Source: PLoS One. 2023 Aug 17;18(8):e0289651. doi: 10.1371/journal.pone.0289651 (PMC10434948; doi:10.1371/journal.pone.0289651)
Supplement: Attachment — Submitted filename: Response to Reviewers.pdf [file pone.0289651.s003.pdf]

Major points:

1. Equations (4) -- (6).

Although the author describes a general framework for the eigenvalue decomposition or the singular value decomposition, it is still unclear how to convert the matrix D into the vector X (= (x_1, x_2, ..., x_N) ?). Furthermore, the definition of the second and the third eigenvectors are insufficient. The order of the corresponding eigenvalues is not defined.

A.)

The transformation of the equation from D to X is shown in equation (4).

L160: $\mathbf{D}^2$ was defined as the matrix of all components of the distance matrix **D** squared, and multiply $\mathbf{D}^2$ by the N × N centralization matrix **J** (=**E**-1/N**1**) from both sides.

L163: **X*** is the centered data matrix and **K** is the inner product N × N matrix obtained from the centered data matrix.

L168: Eigenvalues and eigenvectors are arranged in order of magnitude of the eigenvalues.

2. p6. l175.

The author should understand that E_i does NOT have the physical unit because the matrix V is a unitary matrix, and the eigenvectors are normalized without the physical unit. Therefore, E_i is NOT the "physical" distance.

A.)

Certainly, E_i is not a physical distance. Therefore, "physical" has been removed.

3. p7. ll191--196.

The definition and algorithm of the root were improved and readable. More simply put, is the location of the local minimum of E^-_i the root?

A.)

That's right. In other words, the location of the local minimum E^-_i was set as the end of the DNA loop.

Typos:

1. p2. l23. stoHi-C -> The stoHi-C
2. p4. l105. Fig 1 shows -> Figure 1 shows

A.)

All typos have been corrected.
